# Coronary artery disease as an independent predictor of short-term and long-term outcomes in patients with type-B aortic dissection undergoing thoracic endovascular repair

**DOI:** 10.3389/fcvm.2022.1041706

**Published:** 2022-12-14

**Authors:** Wei Li, Songyuan Luo, Wenhui Lin, Sheng Su, Wenmin Xu, Xiaolu Hu, Yuan Liu, Wenhui Huang, Jianfang Luo, Yingling Zhou

**Affiliations:** ^1^The Second School of Clinical Medicine, Southern Medical University, Guangzhou, China; ^2^Department of Cardiology, Guangdong Cardiovascular Institute, Guangdong Provincial Key Laboratory of Coronary Heart Disease Prevention, Guangdong Provincial People's Hospital, Guangdong Academy of Medical Sciences, Guangzhou, China; ^3^Department of Cardiology, Guangdong Provincial People's Hospital Zhuhai Hospital (Zhuhai Golden Bay Center Hospital), Zhuhai, China; ^4^Department of General Medicine, Guangdong Provincial People's Hospital Zhuhai Hospital (Zhuhai Golden Bay Center Hospital), Zhuhai, China

**Keywords:** coronary artery disease, type B aortic dissection, thoracic endovascular aortic repair, prognosis, predictor

## Abstract

**Background and aims:**

Previous studies reported a high prevalence of concomitant coronary artery disease (CAD) in patients with Type B aortic dissection (TBAD). However, there is too limited data on the impact of CAD on prognosis in patients with TBAD. The present study aimed to assess the short-term and long-term impact of CAD on patients with acute or subacute TBAD undergoing thoracic endovascular aortic repair (TEVAR).

**Methods:**

We retrospectively evaluated 463 patients with acute or subacute TBAD undergoing TEVAR from a prospectively maintained database from 2010 to 2017. CAD was defined before TEVAR by coronary angiography. Multivariable logistic and cox regression analyses were performed to evaluate the relationship between CAD and the short-term as well as long-term outcomes.

**Results:**

According to the results of coronary angiography, the 463 patients were divided into the following two groups: CAD group (*N* = 148), non-CAD group (*N* = 315). In total, 12 (2.6%) in-hospital deaths and 54 (12%) all-cause deaths following a median follow-up of 48.1 months were recorded. Multivariable analysis revealed that CAD was an independent predictor of in-hospital major adverse clinical events (MACE) (odd ratio [OR], 2.33; 95% confidence interval [CI], 1.07–5.08; *p* = 0.033), long-term mortality [hazard ratio (HR), 2.11, 95% CI, 1.19–3.74, *P* = 0.011] and long-term MACE (HR, 1.95, 95% CI, 1.26–3.02, *P* = 0.003). To further clarify the relationship between the severity of CAD and long-term outcomes, we categorized patients into three groups: zero-vessel disease, single-vessel disease and multi-vessel disease. The long-term mortality (9.7 vs. 14.4 vs. 21.2%, *P* = 0.045), and long-term MACE (16.8 vs. 22.2 vs. 40.4%, *P* = 0.001) increased with the number of identified stenosed coronary vessels. Multivariable analysis indicated that, multi-vessel disease was independently associated with long-term mortality (HR, 2.38, 95% CI, 1.16–4.89, *P* = 0.018) and long-term MACE (HR, 2.79, 95% CI, 1.65–4.73, *P* = 0.001), compared with zero-vessel disease.

**Conclusions:**

CAD was associated with short-term and long-term worse outcomes in patients with acute or subacute TBAD undergoing TEVAR. Furthermore, the severity of CAD was also associated with worse long-term prognosis. Therefore, CAD could be considered as a useful independent predictor for pre-TEVAR risk stratification in patients with TBAD.

## Introduction

Type B aortic dissection (TBAD) is considered to be a catastrophic disease with high morbidity and mortality (1). With the development of endovascular interventional technology, thoracic endovascular aortic repair (TEVAR) is considered as valuable strategy for both acute or subacute TBAD (2). However, both short- and long-term postoperative complications and mortality remain high, with an overall mortality of 8.4% in short-term and 25% in long term follow-up (3). Therefore, an accurate preoperative assessment is required to determine high-risk patients for poor prognosis.

Previous clinical studies had reported a variable prevalence of concomitant coronary artery disease (CAD) ranging from 5 to 46% in patients with aortic disease (4–6). Some studies have shown that patients diagnosed with abdominal aortic aneurysm presented a higher prevalence of CAD, which accounts for worse prognosis especially higher perioperative myocardial infarction and mortality in those undergoing aortic procedure (7). In our previous study, we found that the prevalence of CAD in patients older than 50 years with TBAD was relatively high (26.5%) and coronary angiography (CAG) was a safe method to determine concomitant CAD in this specific population before TEVAR (8). However, there is too limited data on the impact of CAD on prognosis in patients with TBAD, especially in those undergoing TEVAR.

Therefore, in this study, we aimed to assess the short-term and long-term impact of CAD on patients with acute or subacute TBAD undergoing TEVAR.

## Methods

### Patient population

**Figure 1:**
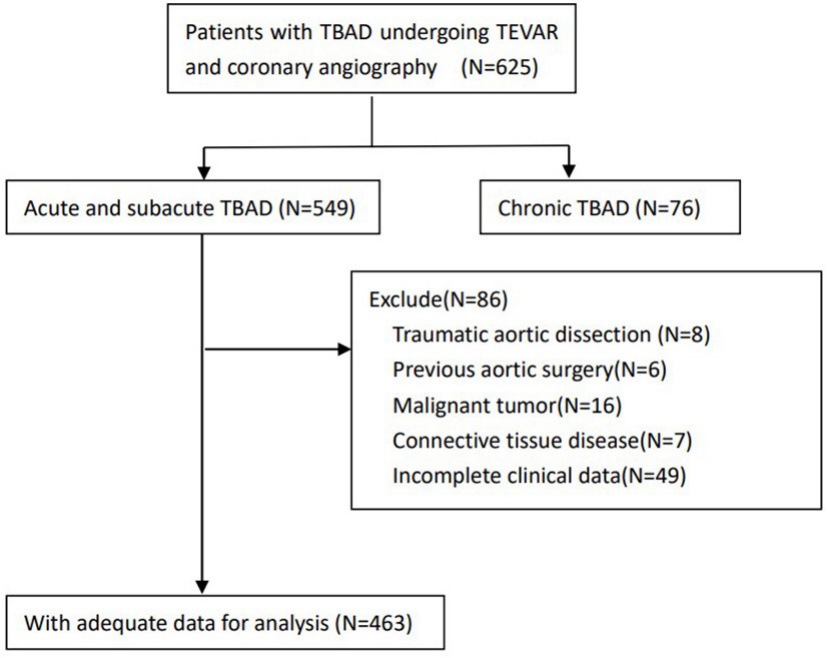
Flow chart demonstrating the inclusion of patients in the study. TBAD, type B aortic dissection; TEVAR, thoracic endovascular aortic repair.

Between January 2010 and December 2017, we retrospectively performed a study of consecutive patients diagnosed with acute or subacute TBAD undergoing TEVAR and coronary angiography in Guangdong Provincial People's Hospital (Guangdong, China). TBAD was diagnosed based on contrast-enhanced computed tomography angiography (CTA) using Stanford classification criteria (2). The exclusion criteria were as follows: (1) traumatic aortic dissection, (2) previous aortic surgery, (3) malignant tumor, (4) connective tissue disease, and (5) incomplete clinical data (Figure 1).This study was approved by the ethics committee of Guangdong Provincial People's Hospital and informed consent was waived due to the retrospective nature of the analysis.

### Diagnosis and management of coronary artery disease

As we described in our previous study (8), the prevalence of CAD in patients older than 50 years with TBAD was as high as 26.5% and CAG was a safe method to determine concomitant CAD. So, CAG was performed routinely in our hospital for patients older than 50 years with TBAD before TEVAR. If Allen test indicating a well-functioning ulnar artery, we routinely use left radial artery as the access for aortography to evaluate the aortic lesion before TEVAR and guide accurate deployment of stent graft. So left radial artery was also considered as the routine access for CAG. Left ulnar artery, left brachial artery were used alternatively for patients we could not get left radial access. Significant stenosis (≥50% lumen diameter stenosis) in major epicardial artery was categorized as CAD. Patients with CAD were further categorized as single-vessel disease or multi-vessel disease according to the number of stenosed vessels. The results of CAG were assessed by two experienced cardiologists. In case of disagreement, a third cardiologist was advised and the majority view was adopted (9).

As recommended by guidelines, all patients diagnosed with CAD received optimal medication treatments, which included antiplatelet drugs, statins, β-blockers, and angiotensin-converting enzyme inhibitors/angiotensin II receptor blockers (10). For patients with TBAD and chronic coronary syndrome (CCS), elective coronary revascularization including percutaneous coronary intervention (PCI) and coronary artery bypass grafting (CABG) was performed after TEVAR according to the assessment of stress myocardial perfusion imaging (MPI) or invasive fractional flow reserve (FFR). For patients with TBAD combined with acute coronary syndrome (ACS), different treatment strategies were adopted according to the risk stratification of ACS. For patients with ST-segment elevation myocardial infarction (STEMI) or very high-risk ACS, TEVAR and PCI were performed simultaneously. Patients with non-very high-risk ACS adopted a strategy of TEVAR first, followed by elective coronary revascularization.

### Thoracic endovascular aortic repair procedure

As recommended by latest guidelines, all patients with TBAD received optimal medication treatments (2, 11). For patients with complicated TBAD, TEVAR was recommended according to latest guidelines (2, 12, 13). For uncomplicated TBAD patients, the indication for TEVAR included the following aspects: (1) primary entry tear diameter more than 10 mm, (2) aortic diameter more than 40 mm, (3) false lumen diameter more than 22 mm, and (4) a patent or partial thrombosed false lumen (14, 15).

The details of the TEVAR procedure in our center have been previously described (14, 16). In brief, the procedures were performed by a multidisciplinary team including interventional cardiologists, cardiothoracic surgeons, anesthetists as well as intensivists. Stent-grafts with the diameter oversized by 5–10% were placed *via* femoral artery access to cover the primary entry tear. For patients with proximal landing zones 1 and 2 or an aberrant right subclavian artery, aortic arch bypass or chimney stent was a supplement to TEVAR, after comprehensive consideration of the patient's aortic anatomy and willingness. The final decision on TEVAR was a consensus reached with the full informed consent of the patients and family.

### Follow-up and data collection

All patients survived in hospital underwent CTA and clinical follow-up at 3, 6, and 12 months and annually thereafter. The information of survival, medication, symptoms, imaging characteristics as well as other relevant conditions were obtained by telephone interviews or outpatient clinic interviews at corresponding intervals. Demographics, comorbidities, medical history, imaging characteristics, laboratory findings as well as follow-up details were recorded retrospectively on an electronic standardized form and analyzed by two researchers independently.

Blood samples were collected from all patients after admission and measured in our hospital's Laboratory Department through established measuring procedures (ISO 9000 Quality Management and Assurance Standards).

### Definition

TBAD that occurred within 14 days of the onset of symptoms was defined as Acute TBAD and TBAD with an elapsed time between 15 and 90 days was defined as subacute TBAD (2). Complicated TBAD was considered with the presence of recurrent or persistent pain, early aortic expansion, uncontrolled hypertension despite full optimal medication treatments, organ malperfusion, and signs of aortic rupture including increasing periaortic, hemothorax, and mediastinal hematoma (2).

Outcomes were reported according to the TBAD reporting standards (13). The primary outcomes of interest were in-hospital mortality and long-term all-cause mortality. And in-hospital major adverse clinical events (MACE) as well as long-term MACE were considered as the secondary outcomes. In-hospital MACE included death, stroke, limb ischemia, visceral ischemia, spinal cord ischemia, and re-intervention. Long-term MACE included all-cause death, acute coronary syndrome (ACS), stroke and re-intervention.

Besides, we also evaluated complications related to coronary procedure including puncture site complications, device-related artery injury, sustained arrhythmias, contrast allergy and coronary complications.

### Statistical analysis

The missing data were interpolated using the mean value in the study as the proportion of missing data for analysis was not >5%. Continuous variables are reported as mean ± standard deviation values or median and interquartile range values. Continuous data was compared using Student's *t*-test of normal distribution or the Mann–Whitney *U*-test for non-normal distribution. Categorical variables are expressed as percentages and compared using Chi-squared analysis or Fisher's exact test. In order to identify the independent predictors of short-term and long-term outcomes, univariate and multivariate logistic and Cox regression analyses were performed. Generally, variables with *P*-value < 0.1 in the univariate analysis or those (*P*-value ≥ 0.1) thought to be clinically important were include in the multivariate analysis using a forward stepwise approach. Survival curves were estimated through the Kaplan-Meier (KM) method. A two-tailed *P*-value < 0.05 was considered significant statistically. All statistical analyses were carried out by using the SPSS 23.0 software (IBM SPSS 23 Inc).

## Results

### Baseline characteristics

A total of 463 acute or subacute TBAD patients undergoing TEVAR were analyzed, including 272 (58.7%) complicated TBAD. The mean age of the total study population was 59.81 ± 8.75 years, and 394 (85.1%) were male. According to coronary angiography, the prevalence of CAD was 32.0% (148/463), including 93 (62.8%) patients with single-vessel disease and 55 (37.2%) patients with multi-vessel disease. For patients with CAD, 1 patient received TEVAR and PCI simultaneously, 21 patients received PCI after TEVAR and 1 patient received CABG after TEVAR.

The 463 patients were divided into the following two groups: CAD group (*N* = 148), non-CAD group (*N* = 315).The baseline characteristics of the two groups are described in Table 1. In the CAD group, patients had older age (61.28 ± 8.51 vs. 59.13 ± 8.79, *P* = 0.014), higher percentage of stroke history (7.4 vs. 3.2%, *P* = 0.040) and chronic kidney disease (CKD) history (10.8 vs. 5.7%, *P* = 0.050) as well as higher preoperative creatinine level (120.99 ± 106.79 vs. 104.20 ± 62.16, *P* = 0.035). Regarding medications at admission, in the CAD group, more antiplatelet drugs (52.7 vs. 26%, *P* = 0.001) and statins (61.2 vs. 42.5%, *P* = 0.001) were used. There was no difference in the gender, hypertension, diabetes mellitus, hyperlipidemia, anemia, smoke, complicated TBAD, acute TBAD, TEVAR with aortic arch bypass, TEVAR with chimney stent and maximum aortic diameter between the two groups.

**Table 1:** Baseline demographics of patients with and without CAD.

	**CAD**	**Non-CAD**	***p***
	**(*N* = 148)**	**(*N* = 315)**	
Age (years)	61.28 ± 8.51	59.13 ± 8.79	**0.014**
Male	132 (89.2)	262 (83.2)	0.090
Hypertension	133 (89.9)	268 (85.1)	0.159
Diabetes	28 (18.9)	41 (13)	0.096
Stroke	11 (7.4)	10 (3.2)	**0.040**
CKD	16 (10.8)	18 (5.7)	**0.050**
Hyperlipidemia	45 (30.4)	73 (23.2)	0.096
Anemia	71 (48.6)	156 (49.8)	0.809
Smoke	72 (48.6)	156 (49.5)	0.861
Complicated TBAD	89 (60.1)	183 (58.1)	0.678
Acute TBAD	91 (61.5)	184 (58.4)	0.530
Maximum aortic diameter (mm)	40.3 ± 8.34	39.68 ± 8.99	0.478
TEVAR with aortic arch bypass	21 (14.2)	64 (20.3)	0.112
TEVAR with chimney stent	21 (14.2)	65 (20.6)	0.096
WBC (10^9^/L)	10.63 ± 3.69	10.27 ± 3.40	0.308
PLT (× 10^9^/L)	223 ± 99.52	215 ± 93.24	0.394
Hemoglobin (g/L)	127.98 ± 16.79	127.34 ± 17.78	0.716
D-Dimer (μg/ml)	3.79 ± 5.08	3.99 ± 12.24	0.850
ALT (U/L)	28.54 ± 44.16	26.83 ± 28.85	0.619
AST (U/L)	30.21 ± 41.66	26.09 ± 17.28	0.261
ALB (g/L)	32.74 ± 4.40	32.90 ± 5.11	0.751
Creatinine (umol/l)	120.99 ± 106.79	104.20 ± 62.16	**0.035**
Cholesterol (mmol/l)	4.38 ± 1.06	4.41 ± 2.02	0.859
Triglyceride (mmol/l)	1.47 ± 0.78	2.35 ± 8.44	0.568
LDL-C (mmol/l)	2.58 ± 0.84	2.62 ± 0.75	0.632
LVEDD (mm)	47.94 ± 5.29	47.65 ± 5.31	0.614
LVESD (mm)	30.77 ± 5.34	29.98 ± 4.94	0.154
LVEF (%)	63.83 ± 6.99	65.14 ± 7.14	0.081
**Medications at admission**			
Antiplatelet drugs	78 (52.7)	82 (26)	**0.001**
ACEI	28 (18.9)	58 (18.4)	0.896
ARB	84 (56.8)	155 (49.2)	0.129
CCB	113 (76.4)	245 (77.8)	0.732
Beta-blockers	140 (94.6)	296 (94.0)	0.789
Statins	90 (61.2)	133 (42.5)	**0.001**

Values are given as number (percentage) or mean ± SD. ACEI, angiotensin-converting enzyme inhibitors; ALB, albumin; ALT, alanine aminotransferase; ARB, angiotensin receptor blockers; AST, aspartate aminotransferase; CKD, chronic kidney disease; CCB, calcium channel blockers; LDL-C, low density lipoprotein cholesterol; LVEDD, left ventricular end diastolic dimension; LVESD, left ventricular end systolic dimension; LVEF, left ventricular ejection fraction; TBAD, type B aortic dissection; TEVAR, thoracic endovascular aortic repair; WBC, white blood cell. Bold value means *P* < 0.05.

### Short-term outcomes

The short-term outcomes are shown in Table 2. In total, 12 (2.6%) in-hospital deaths were recorded, including 9 aortic-related deaths and 1 cardiovascular-related death. There was no difference of in-hospital mortality between the two groups (4.1 vs. 1.9%, *P* = 0.211). During the hospitalization period, 10 patients (2.2%) experienced stroke, 8 patients (1.7%) experienced spinal cord ischemia, 14 patients (3.0%) had limb ischemia, 1 patient (0.2%) had visceral ischemia and 4 patients (0.9%) underwent re-intervention. Of note, the rate of in-hospital MACE was significantly higher in the CAD group than that in the non-CAD group (11.5 vs. 6%, *P* = 0.041).

**Table 2:** Short- and long-term outcomes of patients with and without CAD.

**Outcomes**	**CAD**	**Non-CAD**	***p***
	**(*N* = 148)**	**(*N* = 315)**	
In-hospital MACE	17 (11.5)	19 (6.0)	**0.041**
In-hospital death	6 (4.1)	6 (1.9)	0.211
Aortic-related death	5 (3.4)	4 (1.3)	0.153
Cardiovascular-related death	1 (0.7)	0 (0)	0.320
Stroke	7 (4.7)	3 (1.0)	**0.014**
Spinal cord ischemia	2 (1.4)	6 (2.0)	0.999
Limb ischemia	5 (3.4)	9 (2.9)	0.925
Visceral ischemia	0 (0)	1 (0.3)	0.999
Re-intervention	1 (0.7)	3 (1.0)	0.999
Long-term MACE	41 (28.9)	52 (16.8)	**0.003**
Follow-up death	24 (16.8)	30 (9.7)	**0.029**
Aortic-related death	3 (2.1)	9 (2.9)	0.561
Cardiovascular-related death	6 (4.2)	3 (1.0)	**0.031**
ACS	5 (3.5)	2 (0.6)	**0.034**
Stroke	12 (8.5)	11 (3.6)	**0.028**
Re-intervention	10 (7.0)	15 (4.9)	0.346

ACS, acute coronary syndrome; CAD, coronary artery disease; MACE, major adverse clinical events. Bold value means *P* < 0.05.

Multivariable logistic analysis demonstrated that CAD was an independent predictor of in-hospital MACE (odd ratio [OR], 2.33; 95% confidence interval [CI], 1.07–5.08; *p* = 0.033). Other independent predictors for in-hospital MACE were age, TEVAR with aortic arch bypass and stroke history (Table 3).

**Table 3:** Multivariable logistic regression analyses for in-hospital MACEs.

**Clinical variables**	**OR (95%CI)**	***P***
CAD	2.33 (1.07–5.08)	0.033
Age	1.05 (1.01–1.11)	0.042
TEVAR with aortic arch bypass	4.56 (1.89–10.98)	0.001
Stroke History	6.02 (1.89–19.38)	0.003

Covariates for the multivariable model include age, gender, hypertension, diabetes mellitus, hyperlipidemia, coronary artery disease, stroke, chronic kidney disease, anemia, smoke, complicated TBAD, acute TBAD, TEVAR with aortic arch bypass, TEVAR with chimney stent, maximum aortic diameter, left ventricular ejection fraction, antiplatelet drugs and statins. Variables with a p-value < 0.1 in univariable analysis or those (p ≥ 0.1) thought to be clinically important were entered in the multivariable models. CI, confidence interval; OR, odds ratio; CAD, coronary artery disease; TBAD, type B aortic dissection; TEVAR, thoracic endovascular aortic repair.

There was no significant difference of complications related to coronary procedure between CAD group and non-CAD group (5.4 vs. 4.1%, *P* = 0.538). In the CAD group, 5 patients (3.4%) experienced puncture site complications, 2 patients (1.4%) experienced contrast allergy and 1 patient (0.7%) had coronary spam. In the non-CAD group, 7 patients (2.2%) experienced puncture site complications and 6 patients (1.9%) experienced contrast allergy.

### Long-term outcomes

**Figure 2:**
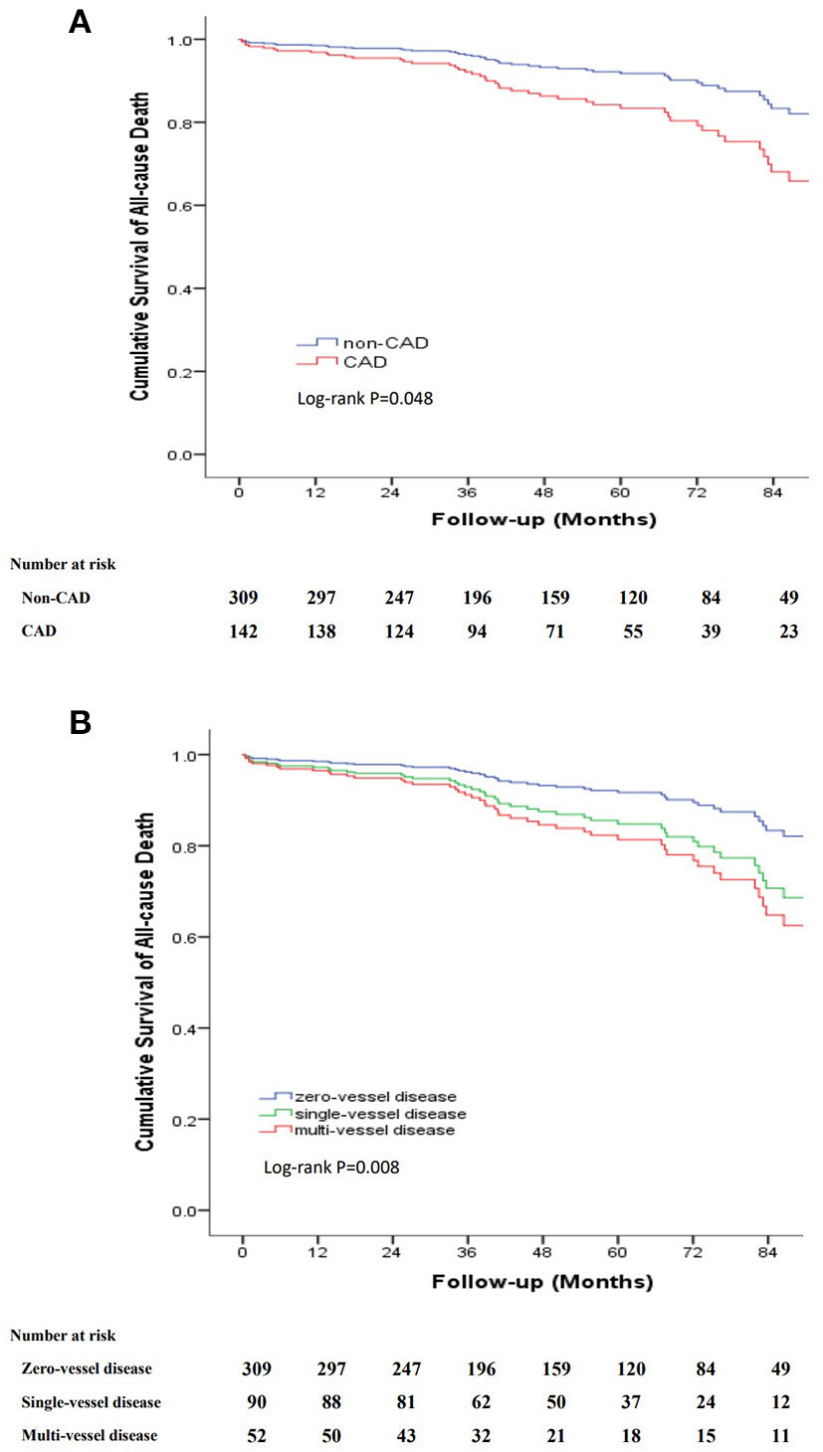
Kaplan-Meier curve for cumulative survival rates of long-term mortality. **(A)** Classified by TBAD patients with and without CAD. **(B)** Classified by the number of identified stenosed coronary vessels.

**Figure 3:**
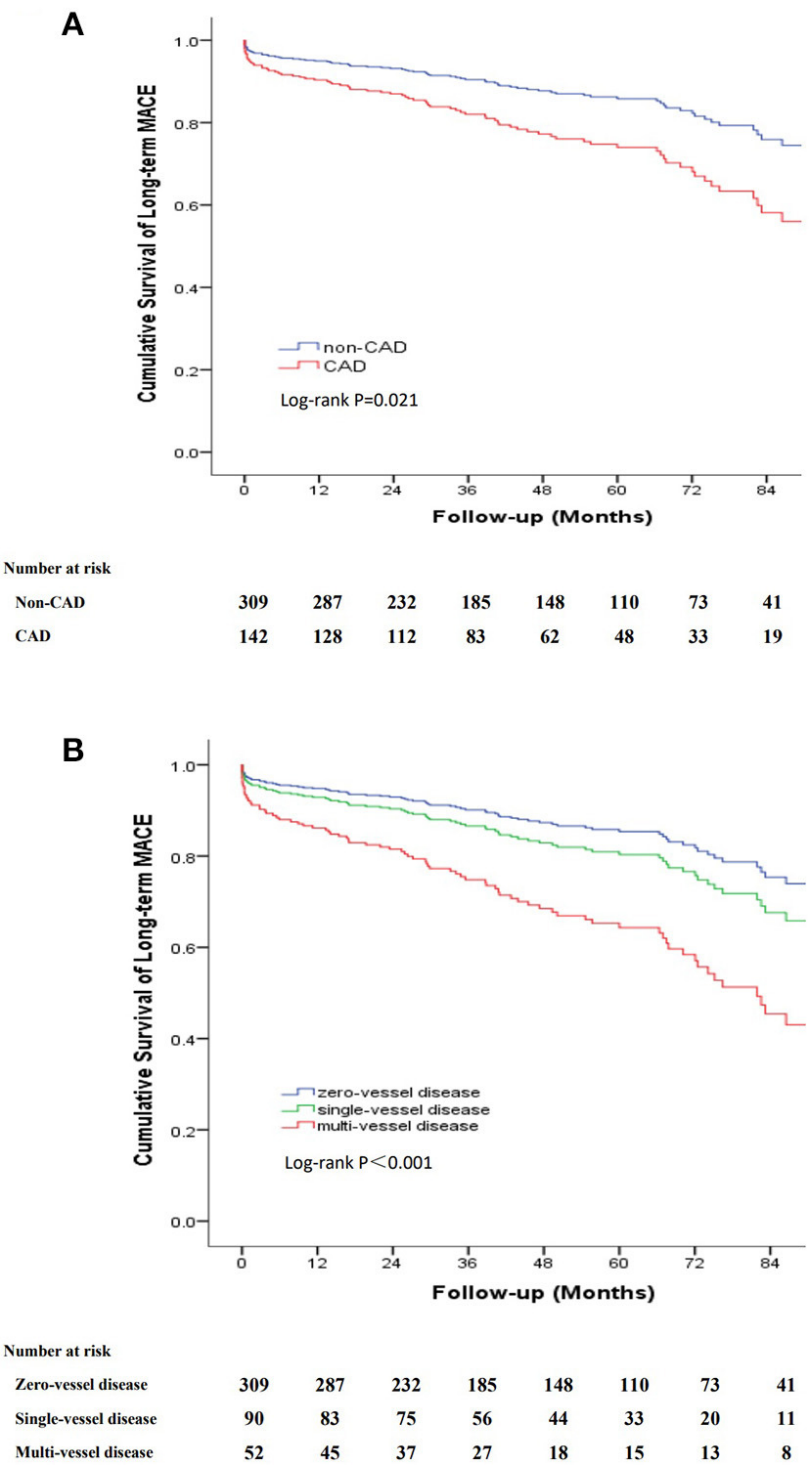
Kaplan-Meier curve for cumulative survival rates of long-term MACE. **(A)** Classified by TBAD patients with and without CAD. **(B)** Classified by the number of identified stenosed coronary vessels.

The long-term outcomes are displayed in Table 2. The mean follow-up period was 48.1 months (interquartile range, 27.2–73.5 months) with a follow-up rate of 92.5%. During the follow-up period, 54 (12%) all-cause deaths were recorded, including 12 aortic-related deaths and 9 cardiovascular-related deaths. Besides, 7 patients (1.6%) suffered acute coronary syndrome, 23 patients (5.1%) suffered stroke and 25 patients (5.5%) needed re-intervention (Table 2). Kaplan–Meier curves indicated that the cumulative long-term mortality was significantly higher in the CAD group than that in the non-CAD group (*P* = 0.048) (Figure 2A). Similarly, significantly higher rate of cumulative long-term MACE was observed in the CAD group (*P* = 0.008) (Figure 3A).

Multivariable Cox regression analysis demonstrated that CAD was associated with long-term mortality independently [hazard ratio (HR), 2.11, 95% CI, 1.19–3.74, *P* = 0.011]. Other independent predictors for long-term mortality included acute TBAD and CKD (Table 4). Similarly, multivariable Cox regression analysis indicated that CAD was associated with long-term MACE independently (HR, 1.96, 95% CI, 1.27–3.03, *P* = 0.002). Other independent predictors for long-term MACE included age, stroke, acute TBAD and TEVAR with aortic arch bypass (Table 4).

**Table 4:** Multivariable cox regression analyses for long-term mortality and MACE.

**Clinical variables**	**HR (95%CI)**	***P***
**Multivariable analyses for long-term mortality**		
CAD	2.11 (1.19–3.74)	0.011
Acute TBAD	0.47 (0.27–0.83)	0.009
CKD	2.38 (1.05–5.41)	0.039
**Multivariable analyses for long-term MACE**		
CAD	1.96 (1.27–3.03)	0.002
Age	1.03 (1.01–1.06)	0.021
Stroke	3.20 (1.45–7.06)	0.004
Acute TBAD	0.53 (0.34–0.82)	0.002
EVAR with aortic arch bypass	1.77 (1.06–2.96)	0.028

Covariates for the multivariable model include age, gender, hypertension, diabetes mellitus, hyperlipidemia, coronary artery disease, stroke, chronic kidney disease, anemia, smoke, complicated TBAD, acute TBAD, TEVAR with aortic arch bypass, TEVAR with chimney stent, maximum aortic diameter, left ventricular ejection fraction. Variables with a p-value < 0.1 in univariable analysis or those (p ≥ 0.1) thought to be clinically important were entered in the multivariable models. CI, confidence interval; HR, hazard ratio; CAD, coronary artery disease; CKD, chronic kidney disease; TBAD, type B aortic dissection; TEVAR, thoracic endovascular aortic repair.

**Figure 4:**
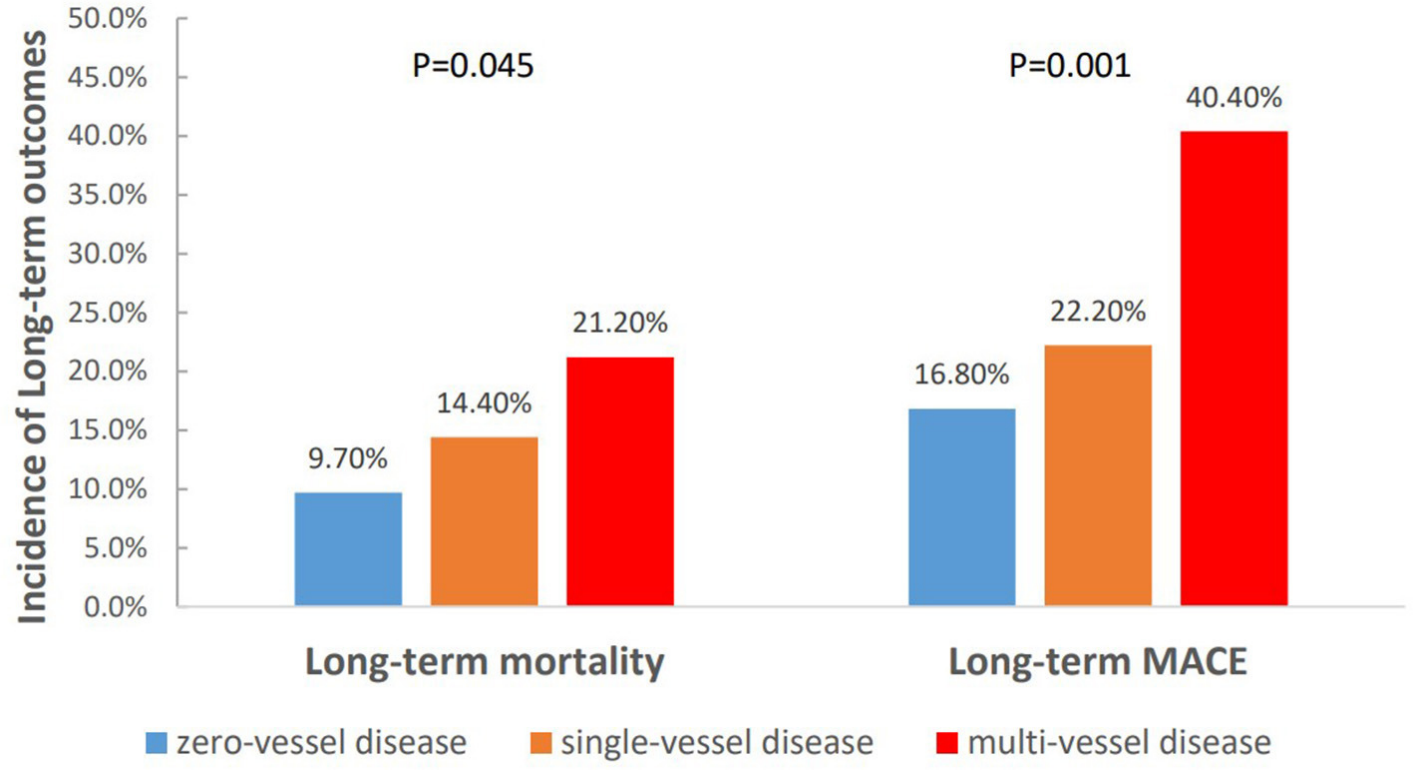
The incidence of long-term outcomes classified by the number of identified stenosed coronary vessels.

To further clarify the relationship between the severity of CAD and long-term outcomes, we categorized patients into three groups according to the number of identified stenosed coronary vessels: zero-vessel disease, single-vessel disease and multi-vessel disease. The incidence of long-term mortality (9.7 vs. 14.4 vs. 21.2%, *P* = 0.045), and long-term MACE (16.8 vs. 22.2 vs. 40.4%, *P* = 0.001) increased with the number of identified stenosed coronary vessels (Figure 4). Kaplan–Meier curves indicated that the cumulative long-term mortality (Figure 2B) and long-term MACE (Figure 3B) among the three groups were significantly different (*P* < 0.001). Multivariable Cox regression analysis indicated that, multi-vessel disease was independently associated with long-term mortality (HR, 2.38, 95% CI, 1.16–4.89, *P* = 0.018) and long-term MACE (HR, 2.79, 95% CI, 1.65–4.73, *P* = 0.001), compared with zero-vessel disease (Supplementary Table 1).

**Figure 5:**
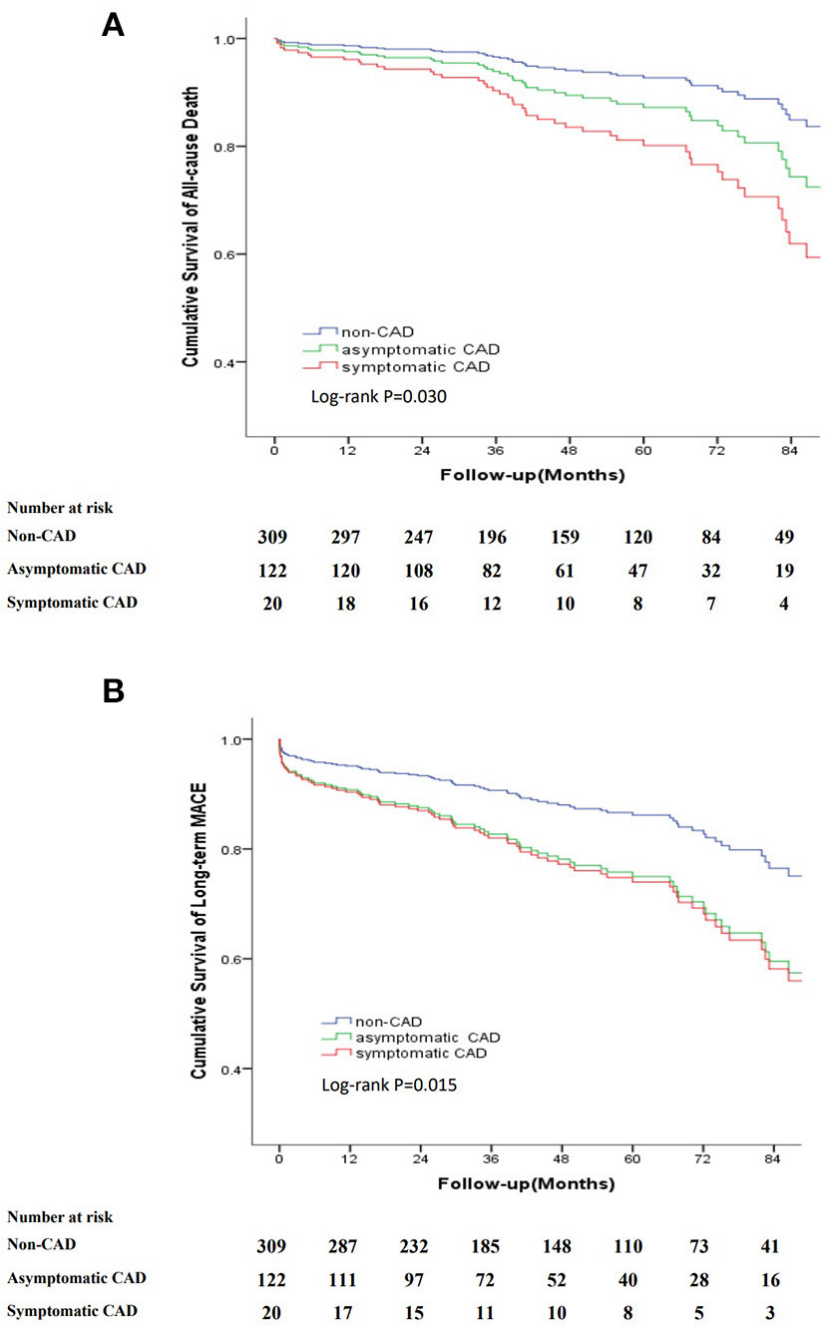
Kaplan-Meier curve for cumulative survival rates of long-term mortality and long-term MACE classified by non-CAD, asymptomatic CAD and symptomatic CAD. **(A)** Kaplan-Meier curve for cumulative survival rates of long-term mortality. **(B)** Kaplan-Meier curve for cumulative survival rates of long-term MACE.

To further analyse the relationship between symptoms associated with CAD and the long-term outcomes, we categorized patients into three groups: non-CAD, asymptomatic CAD and symptomatic CAD. Kaplan–Meier curves indicated that the cumulative long-term mortality was significantly higher in the symptomatic CAD group than that in the asymptomatic CAD group and non-CAD group (*P* = 0.030). Patients with asymptomatic CAD and symptomatic CAD were at a higher risk for long-term MACE than patients without CAD (*P* = 0.015) (Figure 5).

### Subgroup analyses

To further clarify the effect of CAD on the prognosis of different temporal types of TBAD, we stratified the patients by phase of disease (acute vs. subacute). In the acute TBAD subgroup, multivariable regression analyses revealed that CAD remained independently associated with in-hospital MACE (OR, 3.84; 95% CI, 1.43–10.33; *P* = 0.008) and long-term MACE (HR, 1.97, 95% CI, 1.01–3.86, *P* = 0.049). In the subacute TBAD subgroup, multivariable regression analyses revealed that CAD remained independently associated with long-term MACE (HR, 1.85, 95% CI, 1.02–3.48, *P* = 0.048) (Supplementary Table 2).

## Discussion

In the present study, we explored the short-term and long-term impact of CAD on patients with acute or subacute TBAD undergoing TEVAR. We found that CAD was associated with short-term and long-term worse outcomes in patients with acute or subacute TBAD undergoing TEVAR. Furthermore, the long-term mortality and long-term MACE increased with the number of identified stenosed coronary vessels. The cumulative long-term mortality was significantly higher in the symptomatic CAD group than that in the asymptomatic CAD group and non-CAD group. Subgroup analyses indicated that CAD remained independently associated with long-term MACE in both acute and subacute TBAD subgroup. Therefore, CAD could be considered as a useful independent predictor for pre-TEVAR risk stratification in patients with acute or subacute TBAD.

Previous study indicated that atherosclerosis is a principal cause of aortic aneurysm and concomitant CAD. CAD is associated with higher perioperative risk and worse prognosis, which demands accurate diagnosis in an attempt to predict the prognosis and draw out treatment strategy comprehensively (4, 17–19). Although some studies have indicated that AD arise through pathogenic mechanisms that differ from those responsible for atherosclerosis (5, 20), reports from International Registry of Acute Aortic Dissections (IRAD) investigators (21, 22) have indicated that the prevalence of atherosclerosis and hypertension was significantly high in TBAD. AD and CAD also share some risk factors such as hypertension, male gender, smoke and so on. However, there is too limited data on the impact of CAD on prognosis in patients with TBAD, especially in those undergoing TEVAR. To the best of our knowledge, this is the first study to evaluate the short-term and long-term prognosis of patients with TBAD undergoing TEVAR according to presence or absence of concomitant CAD.

Registry studies reported a high prevalence of atherosclerotic disease in patients with aortic disease, including CAD and peripheral artery disease (4). Previous studies indicated that the incidence of CAD in patients with abdominal aortic dissection was 46–71% (5). Naoki Hashiyama's study found that TBAD was significantly more frequently associated with coronary artery atherosclerosis than type A aortic dissection (20). In our previous study, we found that the prevalence of CAD in patients older than 50 years with TBAD was 26.5%. Similarly, in the present study, the prevalence of CAD in patients with TBAD undergoing TEVAR was as high as 32%. This could be explained by the higher exposure to risk factors for CAD in our study population. AD and CAD share some risk factors such as hypertension and male gender (23). In this study, the incidence of hypertension was as high as 86.6% and the proportion of male was up to 85.1%. Apart from this, the high proportion of smoke (49.2%) and high average age (59.81 ± 8.75 years) in our study also increased the likelihood of concomitant CAD in patients with TBAD.

In the present study, CAD was significantly associated with increased risk of in-hospital MACE in patients with TBAD undergoing TEVAR, which was driven primarily by stroke and limb ischemia. The stroke rate (2.2%) and limb ischemia rate (3.0%) in our study were comparable with that reported in previous studies (24–27). Furthermore, our study indicated that TBAD patients with CAD significantly had higher rates of CKD and stroke history as well as higher average age than patients without CAD. These cardiovascular risk factors may reflect the poor pre-operative status and increase overall risk in the perioperative period and long-term follow-up (28, 29).

In our study, patients with CAD significantly had higher rates of CKD than without CAD (10.8 vs. 5.7%, *P* = 0.050). This could be explained by that CAD and CKD share a number of risk factors such as advanced age, male gender, hypertension, and smoking. In previous reports, the incidence of CKD in patients with acute AD was 8.5–10% (30). In a German registry study, the prevalence of CKD was higher among non-survivors in patients with TBAD (23.9 vs. 20% of survivors, *P* = 0.039). Pre-existing CKD was also an independent predictor of mortality in Hoogmoed's study, but not in the IRAD report (31). In the present study, apart from CAD, CKD was independently associated with long-term mortality. There are no data to support a difference in the treatment of TBAD based on the presence of CKD. Early intervention may be a preferred treatment to reduce the duration of ischemia of the visceral organs and kidneys, which is often the only option that offers a reasonable chance of survival for patients with acute TBAD.

Moreover, we have further analyzed the complications related to the coronary procedure and found that there was no significant difference of complications related to coronary procedure between CAD group and non-CAD group. Our results showed satisfactory safety profile of CAG in patients undergoing TEVAR. CAG through radial or brachial artery access theoretically should not produce negative impact on the dissected descending aorta because the diagnostic device has no contact with this pathological segment. This was confirmed by our findings which showed no extension of dissection related to CAG. As far as the additional time is concerned, CAG could be completed within 10 min using single diagnostic catheter through the same pathway established for aortography before TEVAR.

To further clarify the relationship between the severity of CAD and long-term outcomes, our study results showed that the risk of long-term mortality as well as long-term MACE in TBAD patients increased with the number of identified stenosed coronary vessels. Compared to those with zero-vessel disease or single-vessel disease, patients with multi-vessel disease indicated advanced systemic atherosclerosis and subsequent poor prognosis, which was associated with an increased risk of adverse cardiovascular events including myocardial infarction, coronary revascularization, stroke, and/or cardiac death (32). The association between multi-vessel disease and poor prognosis in TBAD patients was found to be independent of traditional risk factors, suggesting a potential benefit for pre-TEVAR risk stratification and management decisions.

There were several limitations in this study. Firstly, our study had selection bias as a single-center retrospective study. Secondly, the study population was patients with acute or subacute TBAD undergoing TEVAR, therefore, our conclusions might not be applicable to patients with TBAD receiving conservative treatment. Thirdly, the impact of perioperative or postoperative therapy for CAD (such as conservative treatment or coronary revascularization) on the clinical outcomes of patients with TBAD were not validated in our study.

In conclusion, the present study identified an association between CAD and short-term and long-term worse outcomes in patients with acute or subacute TBAD undergoing TEVAR. Furthermore, our study also suggested that the severity of CAD was associated with worse long-term prognosis in patients with TBAD undergoing TEVAR. Therefore, CAD could be considered as a useful independent predictor for pre-TEVAR risk stratification in patients with TBAD, especially those with multi-vessel disease or symptomatic CAD.

## Data availability statement

The raw data supporting the conclusions of this article will be made available by the authors, without undue reservation.

## Ethics statement

The studies involving human participants were reviewed and approved by the Ethics Committee of Guangdong Provincial People's Hospital. Written informed consent for participation was not required for this study in accordance with the national legislation and the institutional requirements.

## Author contributions

JL and YZ: research idea and study design. WLi, SL, WLin, and WX: data collection and analysis. WLi, SL, XH, YL, and WH: statistical analysis. WLi and SL: manuscript preparation. All authors: critical revision of manuscript.
