# Supplementary material for: Coronary artery disease as an independent predictor of short-term and long-term outcomes in patients with type-B aortic dissection undergoing thoracic endovascular repair
Source: Front Cardiovasc Med. 2022 Dec 14;9:1041706. doi: 10.3389/fcvm.2022.1041706 (PMC9795049; doi:10.3389/fcvm.2022.1041706)
Supplement: Supplementary file 1 [file Table_1.docx]

**Supplementary Table1. Multivariable cox regression analyses**

**for long-term mortality and MACE**

| Clinical variables | HR(95%CI) | P |
| --- | --- | --- |
| **Multivariable analyses for long-term mortality** |  |  |
| The severity of CAD |  | 0.038 |
| Zero-vessel disease | Reference |  |
| Single-vessel disease* | 1.91（0.95-3.83） | 0.069 |
| Multi-vessel disease* | 2.38（1.16-4.89） | 0.018 |
| Acute TBAD | 0.47（0.27-0.84） | 0.011 |
| CKD | 2.38（1.02-5.33） | 0.044 |
| **Multivariable analyses for long-term MACE** |  |  |
| The severity of CAD |  | 0.002 |
| Zero-vessel disease | Reference |  |
| Single-vessel disease* | 1.38（0.79-2.43） | 0.255 |
| Multi-vessel disease* | 2.79（1.65-4.73） | 0.001 |
| Age | 1.03（1.01-1.06） | 0.035 |
| Stroke | 3.07（1.40-6.74） | 0.005 |
| Acute TBAD | 0.51（0.33-0.79） | 0.003 |

* compared with zero-vessel disease

Covariates for the multivariable model include age, gender, hypertension, diabetes mellitus, hyperlipidemia, the severity of CAD (zero-vessel disease, single-vessel disease or multi-vessel disease), stroke, chronic kidney disease, anemia, smoke, complicated TBAD, acute TBAD, TEVAR with aortic arch bypass, TEVAR with chimney stent, maximum aortic diameter , left ventricular ejection fraction. Variables with a p-value < 0.1 in univariable analysis or those (p≥0.1) thought to be clinically important were entered in the multivariable models. CI, confidence interval; HR, hazard ratio; CAD, coronary artery disease; CKD, chronic kidney disease; TBAD, type B aortic dissection; TEVAR, thoracic endovascular aortic repair.

**Supplementary Table2. Multivariable regression analyses for the impact of CAD on**

**short-term and long-term outcomes in different temporal types of TBAD**

| Clinical variables | Acute TBAD | | Subacute TBAD | |
| --- | --- | --- | --- | --- |
|  | OR/HR(95%CI) | P | OR/HR(95%CI) | P |
| In-hospital MACE | 3.84（1.43–10.33） | 0.008 | 0.58（0.11-3.24） | 0.481 |
| Long-term mortality | 2.12（0.80-5.60） | 0.131 | 1.36(0.60-3.08) | 0.457 |
| Long-term MACE | 1.97 (1.01-3.86） | 0.049 | 1.85（1.02-3.48） | 0.048 |

Covariates for the multivariable model include age, gender, hypertension, diabetes mellitus, hyperlipidemia, coronary artery disease, stroke, chronic kidney disease, anemia, smoke, complicated TBAD, TEVAR with aortic arch bypass, TEVAR with chimney stent, maximum aortic diameter, left ventricular ejection fraction. Variables with a p-value < 0.1 in univariable analysis or those (p≥0.1) thought to be clinically important were entered in the multivariable models. CI, confidence interval; OR, odds ratio; HR, hazard ratio; CAD, coronary artery disease; CKD, chronic kidney disease; TBAD, type B aortic dissection; TEVAR, thoracic endovascular aortic repair.
